# A Comprehensive Model for Diagnosis of Primary Breast Lymphoma Differentiated From Breast Cancer and Prognosis Evaluation of Surgical Treatment

**DOI:** 10.3389/fonc.2022.858696

**Published:** 2022-05-31

**Authors:** Yanan Li, Yan Zhang, Wei Wang, Chong Wei, Danqing Zhao, Wei Zhang

**Affiliations:** Hematology Department, Peking Union Medical College Hospital, Beijing, China

**Keywords:** breast cancer, primary breast lymphoma, ultrasound, mammography, diagnosis

## Abstract

**Background:**

The objective of this work was to discriminate between primary breast lymphoma (PBL) and breast cancer by systematically analyzing clinical characteristics, laboratory examination results, ultrasound features, and mammography findings to establish a diagnostic model for PBL and to analyze the influence of surgical treatment on the prognosis of PBL patients.

**Method:**

We analyzed 20 PBL and 70 breast cancer patients treated during the same period by comparing several characteristics: clinical features, such as age, tumor position, and breast complaints; laboratory examination findings, such as the lactate dehydrogenase (LDH) level, and imaging features such as the maximum diameter, shape, margins, aspect ratio, and calcification of the mass and axillary lymph node involvement. A diagnostic model was then developed using logistic regression analysis. The impact of surgery on the prognosis of PBL patients was assessed through Kaplan–Meier survival analysis.

**Result:**

Breast cancer and PBL could be distinguished based on imaging features, including the maximum diameter, shape, margin, and calcification of the mass, and lymph node involvement (P < 0.05). There were no significant differences between PBL and breast cancer patients in terms of clinical features, or the LDH level. The area under the receiver operating characteristic curve was 0.821. The log-rank test showed that surgery had no significant influence on the prognosis of PBL patients.

**Conclusion:**

Ultrasound and mammography are the most useful methods for detecting malignant breast tumors. Compared with breast cancer tumors, breast lymphoma tumors are larger with a more regular shape and less calcification and are often accompanied by axillary lymph node involvement. Patients with a breast malignancy should not undergo surgical excision without an accurate diagnosis.

## Introduction

Breast cancer has the highest incidence rate and second highest mortality rate in women. There are nearly 279,100 cases of the disease each year, with 42,690 patients dying from breast cancer annually in America (1). Primary breast lymphoma (PBL) is a relatively rare extranodal lymphoma of the breast that accounts for only 0.04%–0.5% of all breast malignancies (2). However, PBL generally exhibits early progression and a poor prognosis. Breast cancer and breast lymphoma are similar with respect to their clinical manifestations, performing as painless breast masses with poor mobility, and imaging examinations reveal nodules with low echo in ultrasound and solitary mass on mammography (3–5). In clinical practice, breast lymphoma is often misdiagnosed as breast cancer or other breast malignancies; however, the treatment of PBL is primarily immunochemotherapy rather than surgery. There are quite a few PBL patients who undergo surgical excision and then suffer from a poor quality of life and have no improvement in prognosis. To date, there is no systematic analysis of the difference between these two malignant cancer types. The purpose of this study was therefore to explore the difference in clinical manifestations and imaging findings between breast cancer and primary breast lymphoma and then to establish a clinical diagnostic model for breast lymphoma. Then, we compared the impact of surgery on the prognosis of primary breast lymphoma patients to provide a diagnostic reference for clinical diagnoses and to help these patients avoid unnecessary radical surgery.

## Materials and methods

### Patients

Our study included all of 20 patients with primary breast lymphoma and 70 patients with breast cancer treated at the same time in Peking Union Medical College Hospital during the period from 2000 to 2020. The inclusion criteria were as follows: 1) age ≥18 years, with a diagnosis of breast cancer or PBL by pathological examination and 2) pathological subtype of diffuse large B-cell lymphoma (DLBCL) for PBL patients and invasive carcinoma for breast cancer patients. The exclusion criteria were as follows: 1) incomplete imaging or laboratory examination data, including the absence of both ultrasound and mammography data, and 2) other types of breast tumors. Detailed patient information is shown in **Table 1**. All PBL patients were followed up *via* telephone until February 1, 2021. The follow-up rate was 90.0%, and the median follow-up time was 36.5 months.

**Table 1:** Clinical and image characteristics between breast cancer and primary breast lymphoma.

	Breast cancer (n = 70)	Primary breast lymphoma (n = 20)	Sig (χ^2^ test)
Age			0.360
<50	27 (38.6%)	10 (50.0%)	
≥50	43 (61.4%)	10 (50.0%)	
Position			0.958
Left	33 (47.1%)	10 (50.0%)	
Right	34 (48.6%)	9 (45.0%)	
Both	3 (4.3%)	1 (5.0%)	
Breast complaints			1.000
Absence	64 (91.4%)	19 (95%)	
Presence	6 (8.6%)	1 (5.0%)	
LDH			0.410
Normal	64 (91.4%)	17 (85.0%)	
More than normal	6 (8.6%)	3 (15.0%)	
Shape			<0.001
Regular	4 (5.7%)	9 (45.0%)	
Irregular	66 (94.3%)	11 (55.0%)	
Margin			<0.001
Circumscribed	4 (5.7%)	8 (40%)	
Others	66 (94.3%)	12 (60%)	
Calcification			0.017
Absence	35 (50%)	16 (80%)	
Presence	35 (50%)	4 (20%)	
Aspect ratio			0.141
<1	51 (72.9%)	18 (90%)	
≥1	19 (27.1%)	2 (10%)	
Lymph node involved			0.007
Absence	48 (68.6%)	7 (35.0%)	
Presence	22 (31.4%)	13 (65.0%)	

### Data Collection

General information was collected from the patients, including sex and age. Clinical manifestations included tumor position, breast-related complaints such as nipple retraction, bloody nipple discharge, and orange peel- or eczema-like skin changes. The main laboratory finding was the serum lactate dehydrogenase (LDH) level. Imaging examinations included color Doppler ultrasound and mammography. Ultrasonography acted as the main modality for assessing the following characteristics: the maximum diameter, shape, margin, and aspect ratio of the mass, and axillary lymph node involvement. We depended on mammography to identify mass calcification. The descriptions of imaging features were based on Breast Imaging Reporting & Data System (BI-RADS) Fifth Edition (2013) (6, 7). The mass shape was classified as 1) regular, including oval and round, or 2) irregular. The mass margin was classified as 1) circumscribed or 2) other, including obscured, microlobulated, indistinct, and spiculated. The aspect ratio was defined as ≥1 when the anteroposterior diameter of any section was greater than or equal to the transverse diameter; otherwise, it was defined as <1. Mass calcification was classified as 1) no or typically benign (rim, round) calcification or 2) suspicious morphology (amorphous, coarse heterogeneous, fine pleomorphic, and fine linear or fine linear branching) morphology.

**Figure 1:**
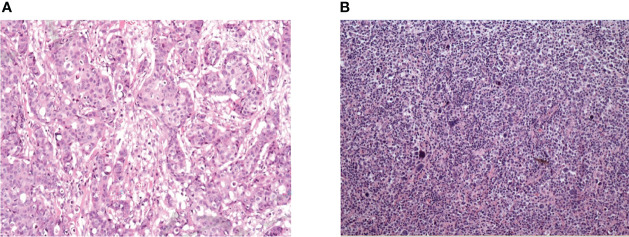
Pathological results of breast cancer and primary breast lymphoma. **(A)** Female, 47, diffuse large B-cell lymphoma. Microscopically, large tumor cells were diffusely infiltrated and homogeneous in shape. Immunohistochemistry: CD20(++), PAX-5(+), CD5(+), Bcl-6(-), CD10(-), Mum-1(-), Ki-67(index 80%). **(B)** Female, 65, invasive breast cancer (non-specific, moderately differentiated), microscopically large tumor cells with invasive growth and acinar distribution, obvious atypia and mitotic visible. Immunohistochemistry: Her-2 (3+), CD10(-), PR(-), CgA(-), Syn(-), P53(+), Ki67 (index 70%).

The gold standard method for diagnosing breast malignancies was pathological examination. Tissues were observed after hematoxylin and eosin (H&E) staining, and the expression of cellular antigens, such as MUM1, Bcl-2, CD10, Bcl-6, CD79a, CD45, CD20, CD3, E-cadherin, ER, PR, and HER-2, was identified through immunohistochemistry (**Figure 1**). In our study, the surgical methods applied to treat breast cancer included classical radical mastectomy, modified radical mastectomy, simple mastectomy, and local mastectomy, excluding breast mass biopsy. The survival of PBL patients was evaluated by the overall survival (OS), defined as the time from the diagnosis of breast lymphoma to the date of death or the date when the follow-up endpoint was reached due to any cause, and progression-free survival (PFS), defined as the time from the beginning of treatment to the date of disease progression or the date when the follow-up endpoint was reached. Examinations of the recorded clinical data were performed by one person. Data verification and regular follow-up visits were used to avoid missing data.

### Data Analysis

SPSS version 20 (IBM) was adopted to process all quantitative data, such as age, tumor site, LDH level, mass shape, mass margin, mass aspect ratio, mass calcification, and axillary lymph node involvement, into dichotomous/trichotomous variables. Then, the χ^2^ test was used to analyze the differences between the PBL and breast cancer patients in terms of age, tumor location, LDH level, and imaging manifestations. T tests or Kolmogorov–Smirnov tests were used to analyze the differences in continuous variables, such as the maximum mass diameter and tumor growth. Taking the characteristics above as independent variables and the type of breast tumor as the dependent variable, bivariate forward stepwise logistic regression was performed. The Wald χ^2^ test was used to estimate the regression parameters, and the likelihood ratio test was used to fit the whole model. Receiver operating characteristic (ROC) curves were used to evaluate the predictive ability of the logistic model. The Kaplan–Meier method was used to analyze the survival outcomes of PBL patients, and the log-rank test was used to calculate the influence of surgery on PFS and OS. All data were analyzed by SPSS 20.0 statistical software, and P < 0.05 was considered statistically significant.

## Results

**Figure 2:**
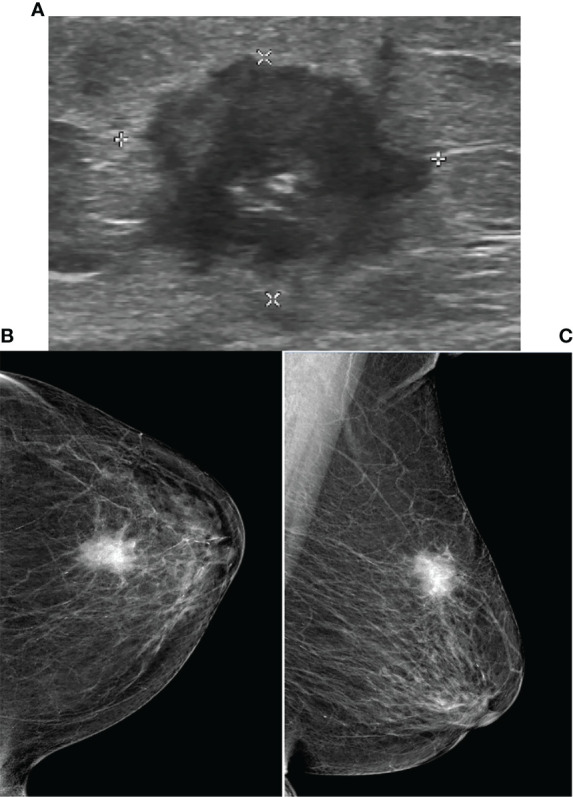
Ultrasound and Mammography images for breast cancer. Legend: Female, 80, invasive carcinoma of left breast. **(A)** showed irregular heterogeneous echo in left breast, spiculated margins. The mammography of CC **(B)** and MLO **(C)** of left breast showed high-density mass, obscure margins, and clustered microcalcifications in the lesion.

**Figure 3:**
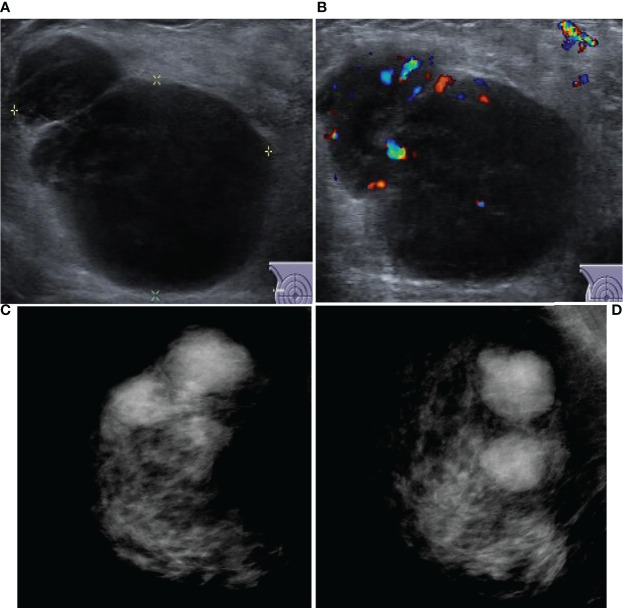
Ultrasound and mammography images for PBL. Legend: Female, 49, right non-Hodgkin breast lymphoma. **(A)** shows irregular hypoecho in the right breast with circumscribed margins. **(B)** CDF1 shows abundant blood flow signals. Right breast mammography CC position **(C)** and MLO position **(D)** image showed high density mass, no micro/macro-calcification.

All 20 patients with PBL in this study had DLBCL; among them, 60% had the germinal center B-cell (GCB) subtype and 40% had the non-GCB subtype. The distribution of BI-RADS categories for PBL patients was as follows: none was categories 0–2; category 3 was 4; category 4 was 13 (4 of category 4A, 7 of category 4B, and 2 of category 4C); and category 5 was 3. Unfortunately, 65% underwent surgery, and all 20 patients later underwent standard immunochemotherapy. All 70 patients with breast cancer were pathologically diagnosed with invasive carcinoma and underwent surgery excision. The distribution of BI-RADS categories for breast cancer was as follows: none was categories 0–2; category 3 was 1; category 4 was 45 (4 of category 4A, 12 of category 4B, and 29 of category 4C); and category 5 was 24. Other clinical and imaging characteristics of the patients are shown in **Table 1** and **Figures 2**, **3**.

Clinical characteristics such as age, tumor location, breast complaints, and LDH level did not differ significantly between the two groups. Among the imaging features, maximum mass diameter (P = 0.007), mass shape (P < 0.001), mass margin (P < 0.001), mass calcification (P = 0.017), and lymph node involvement (P = 0.007) were significantly different between the two groups (**Table 1** and **Supplemental Table 1**). These results indicate that it is difficult to distinguish PBL from breast cancer based on clinical manifestations and that this differentiation mainly depends on imaging examinations.

**Figure 4:**
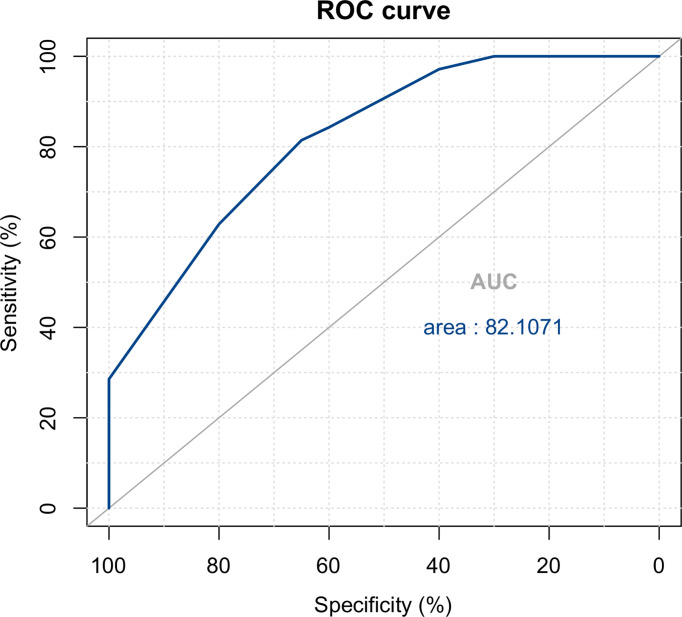
ROC curve for the diagnostic logistic model.

The dichotomous characteristics above were summarized in terms of predictive probability, and finally, three independent risk factors were chosen for inclusion in the logistic model, i.e., mass shape, mass calcification, and lymph node involvement (**Table 2**): Logit(P) = -0.573 + 2.748 × regular shape + 1.296 × no calcification - 1.744 × lymph node involvement. The likelihood ratio test of the above model yielded a statistically significant result (χ^2^ = 27.815, P < 0.001). The Wald χ^2^ test of each regression coefficient showed that the P values of mass shape, mass calcification, and lymph node involvement were less than 0.05. If P = 0.5 was chosen as the threshold, the predictive accuracy was as high as 84.4%, and the sensitivity, specificity, breast lymphoma predictive value, and breast cancer predictive value were 0.814, 0.650, 0.971, and 0.4, respectively. The area under the ROC curve was 0.821 (standard error = 0.053, 95% CI: 0.718–0.924) (**Figure 4**), indicating that the predictive accuracy of the model was high.

**Table 2:** Logistic regression model analysis.

Variables	Regression coefficient	S.E	χ2	P value	OR
Shape	2.748	0.817	11.32	0.001	15.60
Calcification	1.296	0.671	3.73	0.05	3.66
Lymph node involvement	-1.744	0.659	7.00	0.008	0.18
constant	-0.573	0.738	0.60	0.437	0.56

**Figure 5:**
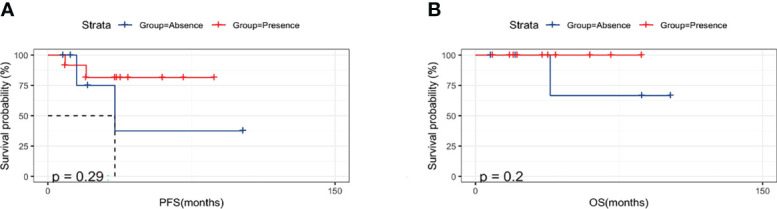
Prognostic influence of operation on primary breast lymphoma.

We then analyzed the impact of surgery on the prognosis of PBL patients. A total of 13 PBL patients (65%) underwent surgical treatment. **Figure 2** shows that surgery had no significant influence on the PFS or OS of these patients, suggesting that surgical treatment did not confer a better prognosis or longer survival time (**Figure 5**). The 10-year PFS and OS rates reached 71% and 87.5%, respectively. With the arrival of the rituximab era, the prognosis of PBL patients has been estimated to be better because of immunochemotherapy. Therefore, while it is not necessary for patients with PBL to undergo surgical treatment, it is vital for doctors to remain vigilant to avoid misdiagnosis.

## Discussion

Breast cancer has high incidence, and aggressive surgery can be used after early detection. However, primary breast lymphoma is rare, and its clinical manifestations are generally similar to those of breast cancer, so clinicians may misdiagnose breast lymphoma as breast cancer. However, the first-line treatment for breast lymphoma is immunochemotherapy rather than surgery (8). A multicenter study showed that mastectomy did not improve the prognosis of PBL patients, and the 5-year OS was 77.3% during the rituximab era (9). Therefore, early differential diagnosis is necessary for the selection of appropriate treatments and to improve quality of life. Although pathology is the gold standard to distinguish breast cancer from breast lymphoma, ultrasound and mammography are the most widely used detection methods in the clinic, playing an important role in the differential diagnosis of breast malignancies. Breast malignancies clinically manifest as palpable and painless masses with poor mobility, which may be accompanied by nipple invagination or discharge. Laboratory tests may show elevated LDH. Previous studies have summarized the ultrasonic characteristics of breast cancer, generally an irregular shape, irregular, spiculated, or microlobulated margins, low echo attenuation, micro/macro-calcification, and the presence of the retraction phenomenon. In contrast, primary breast lymphoma on mammography or ultrasound often shows isolated oval hypoechoic nodules with no obvious obscured margins (10). In addition, PBL has the general characteristics of lymphoma, exhibiting fast growth and a large tumor size. However, no research has systematically and quantitatively differentiated the clinical, laboratory, and imaging manifestations of these two types of cancer. In our research, we evaluated the differences in 10 features (**Table 1** and **Supplemental Table 1**) between the two tumor types and developed a model to differentiate primary breast lymphoma. Significant features with P < 0.05 (including mass maximum diameter, shape, margin, calcification, and lymph node involvement) were found to be primarily related to imaging findings according to the χ^2^ test and t test. Moreover, lymphoma is more likely to invade and metastasize due to the mutation of MYD88 or BCL6 genes and the action of multiple immune cells in the tumor microenvironment (11). There were more regular shapes for breast lymphoma, consistent with previous studies (12, 13). The calcification of breast cancer may be related to the mutation of HER2 or other intrinsic genes (14), or due to the acquisition of osteoblastic characteristics during the process of epithelial-to-mesenchymal transition (EMT), forming matrix vesicles and promoting calcification (15).

Our study is the first to summarize the differences between breast cancer and breast lymphoma and to fit a regression model combining clinical manifestations, laboratory tests, and ultrasound and mammography features, the most widely used modalities in clinical practice. Clinically, the results revealed that at diagnosis, breast lymphoma is typically large in size, with involvement of the surrounding lymph nodes, regular shapes, and no obvious calcification (**Figures 2**, **3**). The area under the ROC curve plotted by the prediction value fitted by logistic analysis was 0.821, supporting the differential diagnosis of breast lymphoma. Therefore, it is necessary for surgeons to perfect breast biopsy when imaging manifestation shows suspected malignancy as more than category 3 (16), rather than an arbitrary diagnosis and surgical resection.

Our research has some limitations, which should be mentioned. First, this was a retrospective study, and the sample size for breast lymphoma was small. Second, we did not analyze the differences in the equipment used between different hospitals. We look forward to performing systematic error correction between instruments and using larger-sample studies in future work.

In conclusion, conventional ultrasound and mammography are useful tools for distinguishing breast cancer from breast lymphoma. The distinguishing characteristics of breast lymphoma included mass maximum diameter, shape, margin, calcification, and lymph node involvement, which are expected to be suggested for clinical differential diagnosis by breast surgeons.

## Data Availability Statement

The raw data supporting the conclusions of this article will be made available by the authors, without undue reservation.

## Ethics Statement

This study was reviewed and approved by the Institutional Review Board of Peking Union Medical College Hospital. Due to the retrospective nature of our research, a waiver of patients consent was obtained from the PUMCH Institutional Review Board. We guaranteed the anonymity and privacy of patient information and the accessed data conformed to the data and privacy regulations of Declaration of Helsinki.

## Author Contributions

YL contributed significantly to the data collection, examination, and analysis and wrote the manuscript. WZ contributed to the conception of the study and the revision of the manuscript. YZ contributed to the research design. WW performed the partial data analysis and was involved in the manuscript preparation. CW helped perform the analysis and provided constructive discussions. DZ helped collect the clinical data and revise the manuscript. All authors read and approved the final version of the manuscript.

## Conflict of Interest

The authors declare that the research was conducted in the absence of any commercial or financial relationships that could be construed as a potential conflict of interest.

## Publisher’s Note

All claims expressed in this article are solely those of the authors and do not necessarily represent those of their affiliated organizations, or those of the publisher, the editors and the reviewers. Any product that may be evaluated in this article, or claim that may be made by its manufacturer, is not guaranteed or endorsed by the publisher.
