# Supplementary material for: A Comprehensive Model for Diagnosis of Primary Breast Lymphoma Differentiated From Breast Cancer and Prognosis Evaluation of Surgical Treatment
Source: Front Oncol. 2022 May 31;12:858696. doi: 10.3389/fonc.2022.858696 (PMC9197495; doi:10.3389/fonc.2022.858696)
Supplement: Supplementary file 1 [file Table_1.docx]

Supplemental table 1 Maximum diameter difference between breast cancer and primary breast lymphoma

|  | N | Mean value | P value |
| --- | --- | --- | --- |
| Primary breast lymphoma  Breast cancer | 20  70 | 3.715±1.001  2.334±0.994 | <0.001 |
